# β-sitosterol interacts with pneumolysin to prevent *Streptococcus pneumoniae* infection

**DOI:** 10.1038/srep17668

**Published:** 2015-12-03

**Authors:** Hongen Li, Xiaoran Zhao, Jianfeng Wang, Yu Dong, Song Meng, Rui Li, Xiaodi Niu, Xuming Deng

**Affiliations:** 1 Key Laboratory of Zoonosis, Ministry of Education, Department of Food Quality and Safety, College of Veterinary Medicine, Jilin University, Changchun, China; 2 Institute of Biophysics, CAS, 15 Datun Road, Chaoyang District, Beijing, 100101, China

## Abstract

Pneumolysin is one of the major virulence factors elaborated by *Streptococcus pneumoniae*; this toxin is a member of the cholesterol-dependent cytolysins. Engagement of cholesterol induces the formation of a multi-subunit complex by pneumolysin that lyses host cells by forming pores on the membrane. Because pneumolysin released by bacteria which have been killed by conventional antibiotics is still active, agents capable of directly attacking the toxin are considered advantageous against antimicrobials in the treatment of *S. pneumoniae* infections. Here we found that the phytosterol, β-sitosterol, effectively protects against cell lysis caused by pneumolysin. This compound interacts with the toxin at Thr459 and Leu460, two sites important for being recognized by its natural ligand, cholesterol. Similar to cholesterol, β-sitosterol induces pneumolysin oligomerization. This compound also protects cells from damage by other cholesterol-dependent toxins. Finally, this compound protects mice against *S. pneumoniae* infection. Thus, β-sitosterol is a candidate for the development of anti-virulence agents against pathogens that rely on cholesterol-dependent toxins for successful infections.

*Streptococcus pneumoniae* is a wide spread bacterial pathogen responsible for many common infections, including, pneumococcal pneumonia, bacteremia, meningitis and otitis media^1^. It is also the major causative agent for community-acquired pneumonia (CAP)^2^. Pneumococcal infection leads to almost 2 million deaths (1 million of which are children under 5 years old)^2^,^3^. The mortality rate of CAP has been relatively consistent despite the medical advances of the past four decades^2^,^3^.

*S. pneumoniae* possesses a number of virulence factors which are essential for its pathogenicity, including hyaluronatelyase (Hyl)^3^, pneumolysin (PLY)^4^, two neuraminidases (NanA and NanB)^4^, the major autolysin (LytA)^4^, choline binding protein A (CbpA)^5^, the pneumococcal surface antigen A (PsaA)^6^, and pneumococcal surface protein A (PspA)^7^. Among these, pneumolysin is a 53 kDa hemolytic protein toxin^8^, which belongs to the cholesterol-dependent cytolysin (CDCs) protein family whose activity requires lipids which present in the membranes of animal cells. This toxin is a crucial factor for acute lung injury (ALI) in lethal *S. pneumoniae* infections and mutants lacking *ply*, the gene coding for the toxin, have dramatically reduced virulence in animal models^9^,^10^. Pneumolysin also has been shown to play important roles not only in the colonization of the nasopharynx and lung tissue by *S. pneumoniae*, but also in the transition from the lung to the cerebral spinal fluid (CSF)^11^.

Antibiotics, the mainstay therapy against *S. pneumoniae* infection, are faced with increasing challenges due to the abundance of strains resistant to commonly used antibiotics such as penicillin, cephalosporins, and macrolides. Further complicating treatment is the release of several toxins by *S. pneumoniae* by the dying bacteria^12^.

Agents that target virulence instead of basic bacterial physiology are considered ideal for the treatment of bacterial infection. Together with the immune system of the host, such agents may be able to resolve the infection without exerting selection pressure that can potentially lead to the development of resistance^13^. In this study we investigated the use of a cohort of steroid alcohols derived from plants to test their effects on toxicity, given the fact that pneumolysin is a cholesterol-dependent toxin. We found that β-sitosterol is able to block the cytolytic activity of pneumolysin. Further studies indicate that this compounds exerts its inhibitory effects by competing with cholesterol for binding to the toxin. We also demonstrate that this compound is able to protect mice from lethal infections by *S. pneumoniae*.

## Results

### Inhibition of the hemolytic activity of pneumolysin by four sterols

**Figure 1:**
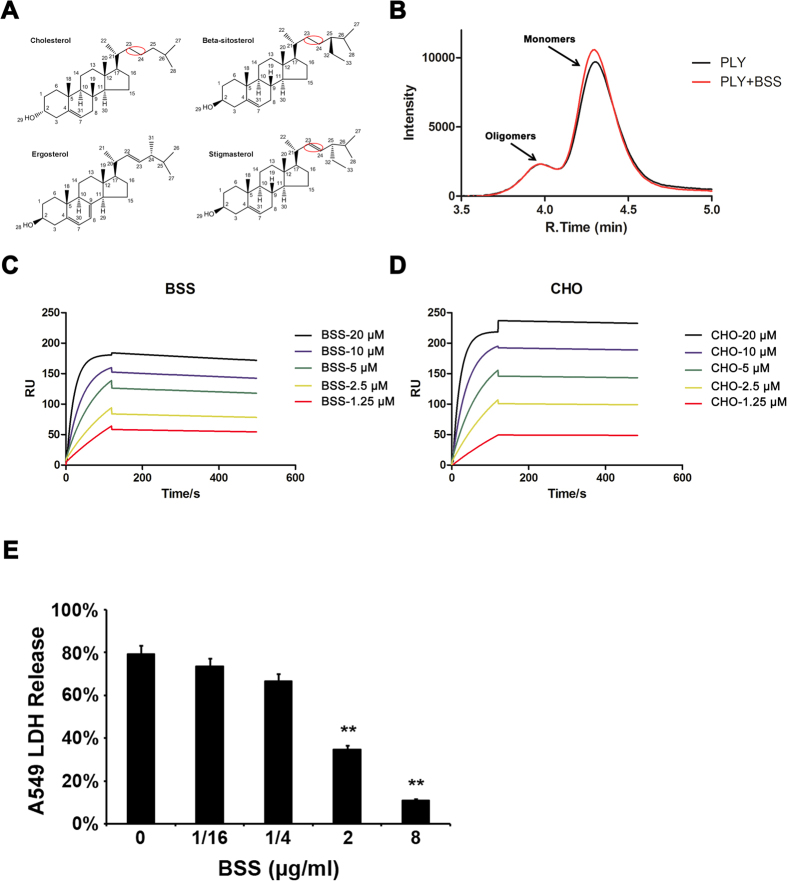
Chemical structures of four sterols and the inhibitory mechanism of β-sitosterol against PLY. (**A**) Chemical structures of four sterols. The red circle indicates the structural differences among cholesterol, β-sitosterol and stigmasterol, which are the critical chemical bonds responsible for the binding to pneumolysin. (**B**) β-sitosterol did not affect the oligoerization of pneumolysin. Purified pneumolysin at a concentration capable of self-assembly (>10 mg/ml) was incubated with β-sitosterol and the mixture was analyzed by high performance liquid chromatograph (HPLC). The toxin eluted in a profile identical to the control which did not receive β-sitosterol. (**C**,**D**) The interactions of pneumolysin with cholesterol or β-sitosterol. Pneumolysin was immobilized on an SPR assay chip and liposomes containing β-sitosterol or cholesterol at the indicated concentrations were used to determine the binding. (**E**) β-sitosterol protects human alveolar epithelial cells from cells injury caused by PLY. A549 cells were treated with the toxin in medium supplemented with different concentrations of β-sitosterol. Cell injury was measured by the release of LDH. The values in the bars represent the means±SD of three independent experiments. *p < 0.05 and **p < 0.01.

To identify novel avenues for treating infections caused by S. *pneumoniae*, we attempted to search for natural compounds which are capable of neutralizing the cytotoxicity of pneumolysin. To this end, we examined the effects of four structurally analogous sterols (Fig. 1A) on pneumolysin-mediated cell lysis by measuring their protection of hemoglobin release induced by the toxin. Despite similarity in structure, these four compounds significantly differ in their ability to block PLY-induced hemolysis (Table 1). Among these, we found that 1 μg of CHO or BSS is sufficient to neutralize the toxicity of 1 μg pneumolysin (Table 1). In contrast, to exert similar protection against 1 μg toxin, 32- and 64-μg of stigmasterol and ergosterol were required, respectively (Table 1). Because BSS exhibited protection at levels similar to CHO, the natural ligand of pneumolysin, we chose to further analyze its mechanism of action.

### BSS has no influence on oligomerization of PLY

Cell lysis by pneumolysin is believed to occur in a two-step process: CHO first attaches to the membrane where it induces pneumolysin oligomerization which then forms pore in the membrane, disrupting its integrity. Spontaneous oligomerization of pneumolysin occurs when its concentration is higher than 10 mg/ml^14^,^15^. Because inhibition of Listeriolysin O oligomerization (another member of the CDC family) by the small molecule compound fisetin leads to abrogation of its toxicity^16^, we thus determined whether β-sitosterol functions by a similar mechanism. Pneumolysin was then incubated with β-sitosterol and the formation of oligomers by the toxin was detected by high performance liquid chromatograph (HPLC). Under experimental conditions, control samples not receiving β-sitosterol were eluted at a retention time of approximately 4.3 min (Fig. 1B). Pre-incubation with BSS at a concentration of 10 nM did not detectably alter the elution profile of the toxin (Fig. 1B). These results suggest that BSS does not interfere with the oligomerization of pneumolysin.

### β-sitosterol has a high affinity for pneumolysin

To further study its mechanism of action, we determined the potential interactions between BSS and pneumolysin. Recombinant pneumolysin was immobilized on a surface plasmon resonance (SPR) chip and liposomes containing β-sitosterol were used as the mobile phase to examine these interactions.

Our results (Table 2) revealed that BSS has a high affinity for pneumolysin, with a *K*_*a*_ of 2.2e3M^−1^s^−1^, similar to that of CHO (1.66e3M^−1^s^−1^) (Fig. 1C,D). However, when the disassociation constant (*K*_*d*_) was measured, we found that the BSS-pneumolysin complex has a *K*_*D*_ of 8.66e^−8^M, which is about 3.65-fold higher than that of the CHO-pneumolysin complex (*K*_*D*_=2.37e^−8^M), indicating that although both molecules bind to pneumolysin with similar affinity, the β-sitosterol -pneumolysin complex is significantly less stable.

### BSS protects human lung cells from injury caused by pneumolysin

In line with its ability to lyse red blood cells, pneumolysin plays a crucial role in cellular damage during the infection of *S. pneumoniae* in cultured cells such as the human alveolar cell line A549^17^. We thus determined the protective effects of BSS on the toxicity of A549 cells by incubating various amounts of the compound with cells treated with pneumolysin and examined cellular damage by measuring the release of lactate dehydrogenase (LDH). Significant protection is achieved when used at a concentration of 2 μg/ml, and the cells were almost completely protected in samples receiving 8 μg/ml BSS (Fig. 1E).

### Residues Thr-459 and Leu-460 are critical for the binding of pneumolysin to β-sitosterol

Previous studies found that besides their participation in the lysis of red blood cells, residues Thr-459 and Leu-460 were also used in PLY cholesterol binding^18^. The structural similarity between BSS and CHO suggests that BSS engages the protein with similar mechanisms. To test this hypothesis, we constructed a pneumolysin mutant in which both Thr-459 and Leu-460 were replaced by a glycine residue. The mutant protein was purified and its interactions with BSS was examined. No interaction was detected between the PLY^T459G · L460G^ mutant and 20 μM BSS (Fig. S1). These results indicate that BSS engages pneumolysin in a similar way to that of CHO.

### Analysis of the interactions between BSS and pneumolysin by molecular modeling

**Figure 2:**
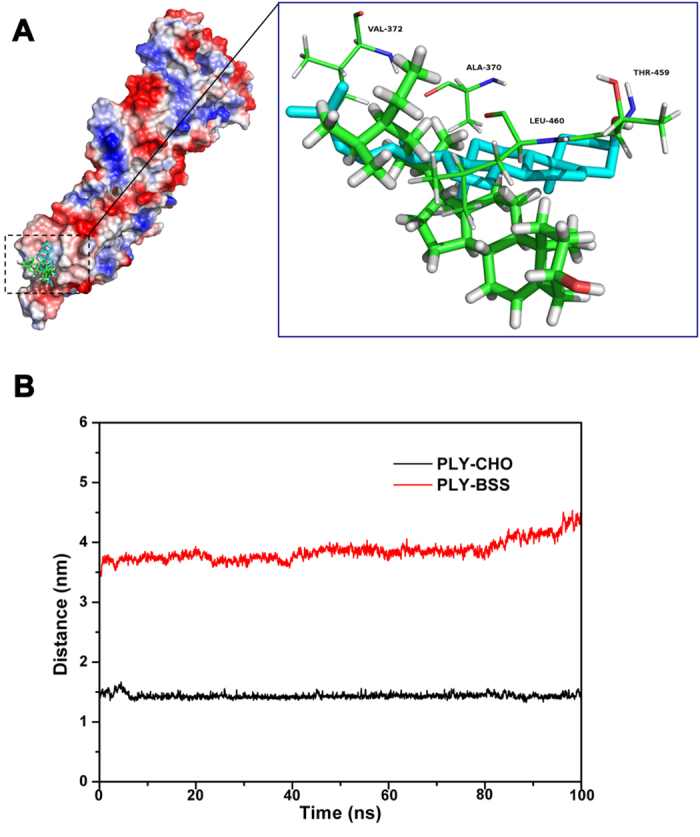
The binding and distance modes of PLY-CHO and PLY-BSS. (**A**) The binding modes of PLY-CHO and PLY-BSS. The binding sites of BSS (molecule in green) with pneumolysin are identical to the binding sites (Val372, Ala370, Leu460 and Thr459) of CHO (molecule in blue), which is the natural receptor of pneumolysin, except for the distance between the respective ligands and Thr459/Leu460. (**B**) Modeling of the distance between ligands, CHO and BSS, and Thr459/Leu460 of pneumolysin as a function of time. The average distance between CHO and Thr459/Leu460 residues is 1.50 nm, and the average distance between BSS and Thr459/Leu460 is 4.02 nm. These results are consistent with those of the binding free energy calculation. The results of the binding free energy calculation show the binding energy of CHO and Thr459/Leu460 is 1.64 and 1.32 kcal/mol and 1.04, 0.52 kcal/mol with BSS. The reason for this is that the distance between BSS and Thr459/Leu460 is longer than that of CHO and Thr459/Leu460.

To explore the mechanism of interaction between pneumolysin and the relevant ligands, we employed molecular dynamics simulations (MD simulation) to analyze the complex between the toxin and CHO and BSS. Overall, the optimized complexes indicated that BSS binds to pneumolysin in a manner highly similar to that of CHO, which is consistent with the experimental results (Fig. 2A). Interestingly, in the predicted BSS-pneumolysin complex, the distance between BSS and Thr459/Leu460 is longer than that seen in the CHO complex (Fig. 2B). The difference is caused by the alkyl chain of C_25_ in BSS, which sterically hindered close interactions between the compound and residues Thr459/Leu460 of pneumolysin. This potential hindrance also provides an explanation to the slightly lower affinity between BSS and the toxin as detected in our SPR analysis (Table 2).

### BSS protects mice from *S. pneumoniae* infection

**Figure 3:**
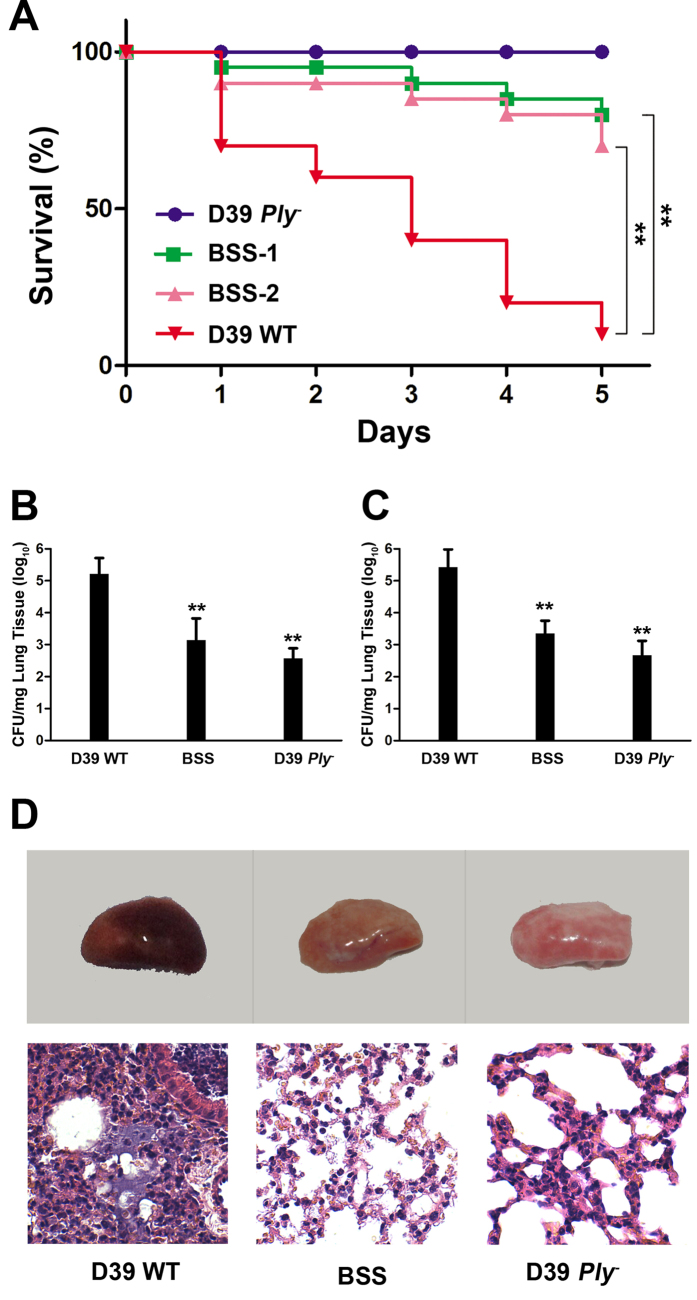
β-sitosterol protects against *S. pneumoniae* infection. (**A**) Survival curves of mice infected with *S. pneumoniae* D39. Mice infected with wild type bacteria were treated at two different time points before infection or postinfection at one hour with β-sitosterol (BSS-1 and BSS-2) or with the solvent (controls) and the mortality of the mice was monitored daily for 5 days. Mice infected with the pneumolysin deficient mutant was established as a control. Note the significant protection achieved by both β-sitosterol concentrations. **p < 0.01. The numbers of bacteria recovered from lungs of differently treated infected mice. Lungs of mice infected for 48 hours were obtained; ground tissues were plated onto bacteriological media to enumerate the bacterial counts. (**B**) The numbers of bacteria recovered from lungs which were treated with BSS before infection for one hour. (**C**) The numbers of bacteria recovered from lungs which were treated with BSS after infection for one hour. All experiments were done in triplicate and similar results were obtained in three independent experiments. **p < 0.01. (**D**) The pathology of lungs of infected mice. Note that in untreated mice infected with D39 WT, the majority of the airspace was obliterated by inflammatory cell infiltrates. Infected mice treated with β-sitosterol showed much less such damage.

We next extended our study of BSS activity by investigating its protective effects against *S. pneumoniae* in infections by employing a mouse disease model. To this end, we intranasally infected groups of mice with *S. pneumoniae* D39 or the pneumolysin-deficient mutant D39-*Ply*^−^. A group of mice infected with wild type bacteria were treated with β-sitosterol at 1 hour after infection. Similar infections were established in mice treated with this compound for 1 hour prior to inoculation. The mortality was monitored at predetermined time points. As expected, no death was observed in mice infected with the toxin-defective bacterial strain (Fig. 3A). In contrast, in the group infected by wild type bacteria, death began to occur 24 hours after infection and approximately 90% of the mice were killed 120 hours after infection (Fig. 3A). Importantly, when administered with 80 mg/kg body weight, mice infected with the wild type bacterium have been protected and the maximal death rate was 30% during the entire experimental duration (Fig. 3A), indicating that BSS can effectively protect animal cells from the lethal infection caused by *S. pneumoniae.* Because the minimal inhibition concentration (MIC) of BSS against *S. pneumonia* is higher than 2048 μg/ml, the blood concentration surely will be significantly lower than this. Thus, the observed protection was not due to direct inhibition of bacterial growth by BSS *in vivo*.

The bacterial burden in the lungs of mice was examined 48 h after infection to evaluate BSS protection. Treatment with BSS prior to or after bacterial challenge led to a marked reduction in bacterial loads. As expectedly, the *ply* mutant failed to replicate during infection (Fig. 3B,C).

We also examined the pathology of the lung from mice 48 hours after infection. In lungs from untreated mice infected with D39 WT, the majority of the airspace was obliterated by inflammatory cell infiltrates. Mice treated with β-sitosterol displayed a marked alleviation of pulmonary inflammation indicated by decreased accumulation of cellular infiltrates in the alveolar space (Fig. 3D).

## Discussion

Owing to antibiotic abuse and difficulty in the discovery of new antibiotics, antibiotic resistance is becoming a severe world crisis in the 21^st^ century. Combating *S. pneumoniae* infections also faced such a crisis. In 1985, Liu *et al* found in South Africa, five clinical isolates of *S. pneumoniae* which displayed tolerance to penicillin^19^. Van-tolerant *S. pneumoniae* (VTSP) has also recently been identified in some countries^20^. To cope with these challenges, various alternative anti-infection strategies have been pursued, and the development of anti-virulence therapeutics was one such strategies^13^. Pneumolysin (one of the major virulence factors) was found in all clinical isolates of *S. pneumoniae*^8^,^9^,^10^. The fact that antibodies specific for pneumolysin provide protection against *S. pneumoniae* infection 21 made the targeting of this toxin an excellent approach to combat infections caused by *S. pneumoniae*.

Cholesterol has been long known to be essential for pore formation by CDC toxins and is their exclusive receptor^22^. Earlier studies suggested that the interactions between cholesterol and pneumolysin are highly specific and that the role of cholesterol cannot be replaced by structurally similar sterols^23^. Our identification of β-sitosterol as an inhibitor comparable to cholesterol points to the potential usefulness of this compound for the abrogation of CDC toxicity. Stigmasterol is also active but at a markedly lower potency.

Unlike fisetin, which inhibits the activity of Listeriolysin O (LLO) by interfering with protein oligomerization^16^, β-sitosterol apparently functions by directly competing for the binding site of its natural ligand. Structurally, stigmasterol has a double bond between the C_23_ and C_24_ atoms, which is absent in cholesterol and β-sitosterol (Fig. 1A). This double bond might cause the distinct differences in the inhibitory activity of these compounds by placing the side chain in stigmasterol after C_23_ on a fixed plane, severely limiting the freedom of rotation allowed in cholesterol and β-sitosterol.

Inhibition of pneumolysin activity affects the ability of *S. pneumoniae* to colonize the lung, and the bacterium failed to migrate to cerebrospinal fluid (CSF) to exacerbate the infection^11^. The effectiveness of β-sitosterol in the prevention of *S. pneumoniae* infection may at least complement the use of antibiotics in treatment, as the latter is known to promote the release of pneumolysin complicating treatment of these infections^12^. For example, sub-lytic concentrations of pneumolysin are capable of impairing the function of the alveolar epithelial-capillary barrier, causing a dysfunction of the sodium transporters required for edema reabsorption^12^. The development of permeability edema can be fatal even after the pathogen has been cleared from the lungs by antibiotic treatment^24^. It will be interesting to determine whether administration of β-sitosterol together with antibiotics will allow for better treatment of infections caused by *S. pneumoniae*.

Our results indicate that the affinity of β-sitosterol to pneumolysin is similar to that of cholesterol but the complexes are not as stable. Such differences may be caused by the branch of C_25_ present in BSS, which constitutes the major structural variation among these molecules (Fig. 1A). This side chain may directly interfere with the binding of β-sitosterol to the toxin or by affecting the positioning of C_27_ and C_28_ so that they cannot properly interact with Thr459 and Leu460. The exact mechanism underlying such differences still awaits further investigation including the determination of the structure of the complex between β-sitosterol and pneumolysin. Such structural information may allow for chemical modification of this compound to produce molecules with higher efficacy.

## Methods

### Bacterial strains and chemicals

*S. pneumoniae* strains D39 and the *ply*-deficient mutant D39 MT^17^ were used in this study. Cholesterol (CHO), β-sitosterol (BSS), ergosterol and stigmasterol were commercially obtained from Sigma-Aldrich (St. Louis, MO, USA). For *in vitro* studies, stock solutions of the sterols at various concentrations were made in a mixture of ethyl acetate and DMSO (v/v = 2:8). For *in vivo* experiments, cytotoxicity assays on cultured cells, Surface Plasmon Resonance (SPR) for interactions with proteins, the effects on protein oligomerization, β-sitosterol or cholesterol was incorporated into liposomes, respectively.

### Liposome Preparation

An established protocol^25^ was used to prepare liposomes. Briefly, 1-palmitoyl-2-oleoyl-sn-glycero-3-phosphocholine (POPC) procured from Avanti Polar Lipids^25^ and β-sitosterol or cholesterol were mixed at a molar ratio of 45:55 in chloroform. The solvent was removed with a stream of argon or nitrogen at 40 °C, and the mixture was further dried under vacuum for 3 hours. 500-μl of the mixture was added to the dried lipids and the suspension was mixed by vortexing and sonication in a water bath for 5 min to ensure that the lipid was fully hydrated. The suspended mixture was then passed 21 times in 0.5 mL increments through an Avestin Inc (Ottawa, ON) liposome extruder to generate liposomes. The liposomes were stored at 4 °C and used within 5 days of preparation.

### Cloning, expression and purification of wild-type pneumolysin

The coding sequence of the *ply* gene was amplified from genomic DNA of *S. pneumoniae* D39 with primers (forward 5′-GCTGGATCCCATATGGCAA ATAAAGCAGT-3′ and reverse 5′-CTGCTCGAGCTAGTCATTTTCTACCTTATC-3′), digested with *BamHI* and *XhoI* then cloned into similarly digested pET28a to yield pET28a-PLY. This construct was transformed into *Escherichia coli* BL21 (DE3) cells for protein expression. The cells were grown at 37 °C in LB broth to a density of OD_600_ = 0.6 and IPTG was added to a final concentration of 0.2 mM IPTG. After induction at 16 °C for 18 hrs, the cells were harvested by centrifugation. To purify the protein, cells from 4 liters of culture were resuspended in 200 ml PBS and lysed by sonication. The cell lysate was cleared by centrifugation at 30,700*g* for 30 min. The supernatant was loaded onto a Ni^2+^–NTA agarose column, which had been equilibrated with five column volumes of PBS. The binding was allowed to proceed for 60 min by gentle rocking at 16 ^o^C. The matrix with bound protein was washed with 10× column volumes of a washing buffer (20 mM Tris pH 8.0, 20 mM imidazole 300 mM NaCl). The His_6_-tagged protein was eluted with five column volumes of an elution buffer (20 mM Tris-HCl, 300 mM imidazole and 300 mM NaCl). The eluted protein was concentrated and desalted using Millipore Amicon filters (30 kDa molecular-weight cutoff). The purity of the protein in PBS was analyzed on SDS–PAGE. Pneumolysin mutants were similarly purified.

### Hemolysis assay

10-μl of purified pneumolysin (100 μg/ml) was incubated in microtiter plates with serially diluted concentrations of the testing sterols at 37 °C for 15 min. A volume of 50 μl (5 × 10^6^ cell/ml) defibrinated sheep blood in PBS was added to the wells and the final volume of the reaction adjusted to 1 ml with PBS. The reactions were incubated at 37 °C for 25 min. Reactions that received 10-μl 1% Triton X-100 and PBS were used as positive and negative controls, respectively.

### Bacteria culture

*S. pneumoniae* strains D39 and the *ply*-deficient mutant D39 MT were grown in Todd-Hewitt broth (THB)+2% yeast extract (THY media). Bacteria were stored in glycerol at −70 °C and thawed at room temperature to inoculate fresh liquid THY medium. Bacteria were grown overnight to stationary phase at 37 °C in a 5% CO_2_ incubator. These bacterial cells were then diluted to appropriate concentrations.

### Oligomerization analysis

5 nM purified pneumolysin was diluted to 0.4 mM (20 mg/ml) and mixed with 10 nM β-sitosterol-rich liposomes (a total volume of 15 μl) and incubated at 37 °C for 1 hr. The mixture was injected into an HPLC system (DGU-20A5, SHIMADZU CORPORATION, Japan) equipped with a Nanofilm SEC-250 column (Sepax Technologies, Inc. USA). The flow rate of the mobile phase was set at 0.5 ml/min. Results were plotted with Prism software (GraphPad Software, Inc.). An identical reaction without the addition of β-sitosterol-rich liposomes was established as the untreated control.

### Site-directed mutagenesis of *ply*

Mutations in targeted residues were introduced into the *ply* gene by using the QuickChange site-directed mutagenesis kit (Stratagene, La Jolla, CA, USA). The mutations T459G and L460G were introduced in the *ply* gene in the plasmid pET28a-PLY. The primer pairs used to introduce these two mutations were: T459G-PLY forward, 5′-TCTATTTGGGGAACAGGTCTCTATCCTCAG-3′, reverse, 5′-ACCTGTTCCCCAA ATAGAAATCGTCCGCTT-3′. For L460G-PLY forward, 5′- ATTTGGGGAACAA CTGGCTATCCTCAGGTA-3′ reverse, 5′-GCCAGTTGTTCCCCAAATAGAAATCG TCCG-3′. The modified codons were underlined in each primer sequence.

### Surface plasmon resonance (SPR) analysis

The affinity and kinetics of pneumolysin, its mutants and cholesterol or β-sitosterol (both as liposome) were measured by SPR at 25 °C on a BIAcore^®^ 3000 using CM5 chips. Pneumolysin or its T459G/L460G mutant was dissolved in 10 mM sodium acetate (pH 4.0) and immobilized on the CM5 chip with 1000 response units (RU) at a flow rate of 10 μL/min. Liposomes containing cholesterol or β-sitosterol were serially diluted in PBST buffer (PBS containing 0.005% Tween 20) to concentrations ranging from 20 μM to 1.25 μM. Each of the five concentrations used was injected at a flow rate of 30 μL/min for 2 min; for dissociation, the flow rate was set at 30 μL/min for 6 min. To regenerate channels, 40 mM β-Octyl glucopyranoside (Sigma-Aldrich St. Louis, MO, USA) was injected for 90 sec at a flow rate of 20 μL/min, followed by injection of PBST buffer for 90 s until the RU reached the original reading. All injections were performed at 25 °C. The data was fitted with a 1:1 binding model using BIA evaluation 4.1. Figures were made using Prism (GraphPad Software, Inc.).

### Molecular modeling

Molecular modeling of the interactions between pneumolysin and cholesterol or β-sitosterol was performed as described previously^16^,^26^,^27^. The binding free energy between PLY and ligands was calculated by the Molecular Mechanis/Poisson-Boltzman Surface Area (MM-PBSA) method^28^ supplied with Amber 10 package^29^. Then, the interaction between inhibitors and each residue in the binding site of PLY was analyzed by using the MM-PBSA decomposition process^30^. The binding of each ligand-residue pair includes three categories: the Van der Waals contribution (ΔEvdw), the electrostatic contribution (ΔEele), and the salvation contribution (ΔEsol).

### Cytotoxicity assays

A549 human lung epithelial cells (ATCC CCL185) were cultured in DMEM medium supplemented with 10% fetal bovine serum (Invitrogen, CA, USA). The cells were seeded in 96-well plates at a density of 1.5 × 10^4^ cells per well. 20 μg of Ply was added to cell cultures containing β-sitosterol at different concentrations. After incubation at 37 °C for 4 h, cell viability was measured by determining extracellular LDH using the Cytotoxicity Detection Kit (LDH) (Roche, Basel, Switzerland) according to the manufacturer’s instructions. Briefly, Ply of indicated concentrations was added together with BSS at the testing concentrations to the cells. After incubation at 37 °C, 5% CO_2_ for 4 hours, culture supernatant was collected by centrifugation and the LDH activity was measured with a microplate reader (TECAN, Austria). The rates of lysis for the treatment were calculated by dividing the readings to that of Triton X-100 treated samples, in which the cellular LDH was completely released.

### Mouse model of intranasal lung infection

Animal experiments were approved by and conducted in accordance with the guidelines of the Animal Care and Use Committee of Jilin University. 8-week old male C57BL/6 mice were obtained from the Experimental Animal Center of Jilin University.

For lung infection, mice were anesthetized intraperitoneally with ketamine and xylazine and then intranasally infected with a dose of 5 × 10^6^ CFU of *S. pneumoniae* strain D39 in 50-μl PBS. The bacteria were applied atraumatically to the tip of the left nose and were involuntarily inhaled^31^. For mice groups treated with β-sitosterol, mice were administered with 100-μl β-sitosterol rich-liposomes subcutaneously 1 hour before or after infection with *S. pneumoniae*, with additional doses given at 4-hour intervals for 48 hours. Each experimental group contained 20 mice with deaths were recorded at 120 hours at a 24-hour interval.

The bacterial burden in lung tissue samples was evaluated at 48 h postinfection by plating appropriately diluted tissue homogenate on blood agar. Bacteria were enumerated after 24 h incubation.

### Statistical analysis

For mortality studies, statistical analysis was performed with the Fisher’s exact test; results in LDH release were analyzed using the two-tailed Student *t* test.

## Additional Information

**How to cite this article**: Li, H. *et al.* ß-sitosterol interacts with pneumolysin to prevent *Streptococcus pneumoniae* infection. *Sci. Rep.* **5**, 17668; doi: 10.1038/srep17668 (2015).

## Supplementary Material

Supplementary Information

## Figures and Tables

**Table 1:** The inhibitory effects of compounds tested in this study against PLY.

	1^a^	8	16	32	64	>1024
Cholesterol	100%^b^	100%	100%	100%	100%	100%
β-sitosterol	100%	100%	100%	100%	100%	100%
Stigmasterol	0%	33.32 ± 1.35%	88.35 ± 1.12%	100%	100%	100%
Ergosterol	0%	10.19 ± 1.73%	51.75 ± 1.97%	78.21 ± 3.29%	100%	100%
Lanosterol	0%	0%	0%	0%	0%	0%
Peimine	0%	0%	0%	0%	0%	0%
Peiminine	0%	0%	0%	0%	0%	0%

^a^The unit for the compounds used is μg/ml.

^b^The rates of protection were calculated by dividing the OD_543_ values to the values obtained by osmatic lysis of sheep red blood cells.

**Table 2:** Interacting affinities of CHO and BSS to pneumolysin measured by SPR

sterol	*K*_a_(M^−1^s^−1^)	*K*_d_(s^−1^)	*K*_A_(M^−1^)	*K*_D_(M)
CHO	1.66e3	3.93e-5	4.23e7	2.37e-8
BSS	2.2e3	1.91e-4	1.16e7	8.66e-8
