# Supplementary material for: β-sitosterol interacts with pneumolysin to prevent Streptococcus pneumoniae infection
Source: Sci Rep. 2015 Dec 3;5:17668. doi: 10.1038/srep17668 (PMC4668377; doi:10.1038/srep17668)
Supplement: Supplementary Information [file srep17668-s1.pdf]

β-sitosterol interacts with pneumolysin to prevent *Streptococcus pneumoniae* infection

Hongen Li[1a], Xiaoran Zhao[1a], Jianfeng Wang[1], Yu Dong[2], Song Meng[1], Rui Li[1], Xiaodi Niu[1#], Xuming Deng[1#]

[1] Key Laboratory of Zoonosis, Ministry of Education, Department of Food Quality and Safety, College of Veterinary Medicine, Jilin University, Changchun, China,

[2] Institute of Biophysics，CAS

[a] These authors contributed equally to this work.

[#]Correspondence and requests for materials should be addressed to X.D.N. (niuxd@jlu.edu.cn) or X.M.D. (dengxm@jlu.edu.cn).

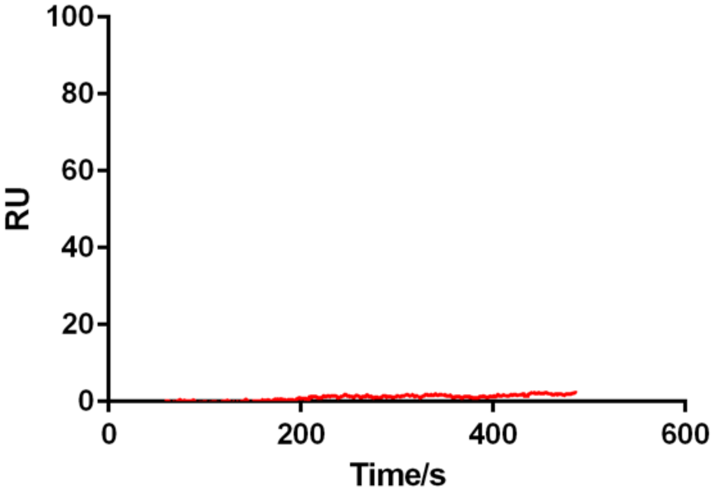

Figure S1. The interactions of mutant pneumolysin with β-sitosterol. Mutant pneumolysin was immobilized on an SPR assay chip and liposomes containing β-sitosterol at the 20 μM concentration was used to determine the binding.
